# Concentration, sources and risk assessment of PAHs in bottom sediments

**DOI:** 10.1007/s11356-017-9944-y

**Published:** 2017-08-21

**Authors:** Agnieszka Baran, Marek Tarnawski, Krzysztof Urbański, Agnieszka Klimkowicz-Pawlas, Iwona Spałek

**Affiliations:** 1 Department of Agricultural and Environmental Chemistry, University of Agriculture in Krakow, Al. Mickiewicza 21, 31-120 Krakow, Poland; 2 Department of Hydraulic Engineering and Geotechnics, University of Agriculture in Krakow, Al. Mickiewicza 24/28, 30-059 Krakow, Poland; 3 Faculty of Mining Surveying and Environment Engineering, Department of Environmental Management and Protection, AGH University of Science and Technology, Al. Mickiewicza 30, 30-059 Krakow, Poland; 4 Department of Soil Science Erosion and Land Protection, Institute of Soil Science and Plant Cultivation—State Research Institute, ul. Czartoryskich 8, Pulawy, Poland

**Keywords:** Bottom sediments, PAHs, Source, Ecological risk assessment, PCA

## Abstract

The aims of the study were to investigate the concentration, sources and ecological risk of PAHs (polycyclic aromatic hydrocarbons) in bottom sediments collected from nine reservoirs located in south-eastern Poland. The concentration of ∑PAHs in sediments ranged from 150 to 33,900 μg kg^−1^. The total PAH concentration in the bottom sediments was arranged in the following order: Rybnik > Rzeszów > Brzóza Królewska > Brzóza Stadnicka > Besko > Chechło > Ożanna > Głuchów > Narożniki. BAP was the major compound in sediments from the Besko, Brzóza Stadnicka and Rzeszów reservoirs; FLT in the sediments from the Rybnik, Narożniki, Ożanna and Brzóza Królewska reservoirs; and FLN from the Głuchów and Chechło reservoirs. The major inputs of PAHs were of pyrolytic origin. However, petrogenic sources of PAHs occurred especially in the Chechło and Głuchów reservoirs. The ecological risk assessment indicated that non-adverse effects on the benthic fauna may occur for sediments from the Głuchów, Narozniki and Ożanna reservoirs, while slightly adverse effects were found for sediments from the Brzóza Królewska, Besko, Brzóza Stadnicka and Chechło reservoirs. The other sediments showed moderate (Rzeszów reservoirs) and strong effect (Rybnik reservoir) on biological communities. Individual PAHs such as NAP, PHE, FLT, PYR, BAA, CHR and BAP in sediments from the Rybnik reservoir and BAP in sediments from the Rzeszów reservoirs indicated a higher possibility of occurrence of an adverse ecological effect. PCA analysis found slight difference between the reservoirs in the profile of variable PAHs. Only the sediments from the Rybnik and Chechło reservoirs differ considerably from this grouping.

## Introduction

Polycyclic aromatic hydrocarbons (PAHs) are composed of two or more fused aromatic rings and belong to persistent organic pollutants (Chen et al. 2015; Sukhdhane et al. 2015; Wang et al. 2016). PAHs occur in the environment as complex multicomponent mixtures which are both natural and anthropogenic (Maliszewska-Kordybach et al. 2009; Lubecki and Kowalewska 2010; Klimkowicz-Pawlas et al. 2016). PAHs are ubiquitous organic contaminants derived mainly from incomplete combustion of organic materials and natural processes (Savinov et al. 2000; Wang et al. 2013; Abdel-Shafy and Mansour 2016). There are three major types of PAHs, which differ in their origin: petrogenic, biogenic and pyrogenic (Dahle et al. 2003). Normally, pyrogenic PAHs are mainly detected in incomplete combustion of organic compounds, such as fossil fuels (heating oil, coal, grass and wood combustion, vehicle emissions, waste tire) (Yan et al. 2009; Liu et al. 2009; Khairy et al. 2009; Tavakoly Sany et al. 2014; Wang et al. 2016). PAHs of petrogenic origin are related to petroleum, including crude and fuel oil, oil spills and oil refined products (Dahle et al. 2003). PAHs from pyrogenic sources are believed to be more thermodynamically stable and toxic than from heterogeneous sources due to their high concentration of non-alkylated PAHs (Tavakoly Sany et al. 2014). Biogenic PAHs can be produced biologically, e.g. they can be synthesized by certain plants, fungi and bacteria or formed during the degradation of organic matter (Abdel-Shafy and Mansour 2016). PAHs in aquatic environments are mainly introduced by dry and wet atmospheric deposition, surface and roadway runoff, storm, municipal and industrial effluents and shipping (Dmitruk et al. 2008; Liu et al. 2009; Kapen et al. 2013). The main sources of atmospheric PAHs are a direct result of incomplete combustion or pyrolysis under reduced conditions of mineral fuel, wood, paper, other hydrocarbons and vehicle emissions. These compounds have low water solubility, less volatility, high lipid solubility and high persistence. Due to their properties, PAHs present in water reservoirs tend to accumulate in sediments, which will have a long-term impact on benthic organisms (Wolska et al. 2003; Qiao et al. 2006; Nasher et al. 2013; Tavakoly Sany et al. 2014; Li et al. 2015; Sukhdhane et al. 2015; Zhonghua et al. 2016). PAHs also can be bioaccumulated through the food chain, and some PAHs are known to be toxic, mutagenic and carcinogenic to humans (Yan et al. 2009; Li et al. 2015; Wang et al. 2016). Therefore, accumulation of PAHs in sediments has received much attention. Selected PAHs have been recognized as high priority pollutants by many conventions, environmental organizations and legislations, such as the Helsinki Convention, the Water Framework Directive, the United States Environmental Protection Agency and the United Nations Environment Programme. The bottom sediments play an important role as a sink for many persistent organic pollutants, and they may pose a risk to the aquatic environment after dredging and disposal on land (Hiller et al. 2009; Urbaniak et al. 2015). To better predict environmental risks associated with PAHs, it is necessary to first determine the concentration and sources of PAHs. Moreover, making decision about the deposition of sediments on land or their agricultural utilization must necessarily include establishing PAH concentration in sediments. To date, however, there have been few studies on PAH concentration in sediments in Polish water reservoirs.

The aims of the study were as follows: (1) to investigate the concentration of PAHs in bottom sediments collected from nine reservoirs located in south-eastern Poland; (2) to distinguish the possible sources of PAHs with diagnostic ratios; (3) to evaluate the potential ecological risk of PAHs; and (4) to analyze the relationship between PAHs in sediments.

## Material and methods

### Study area

**Fig. 1:**
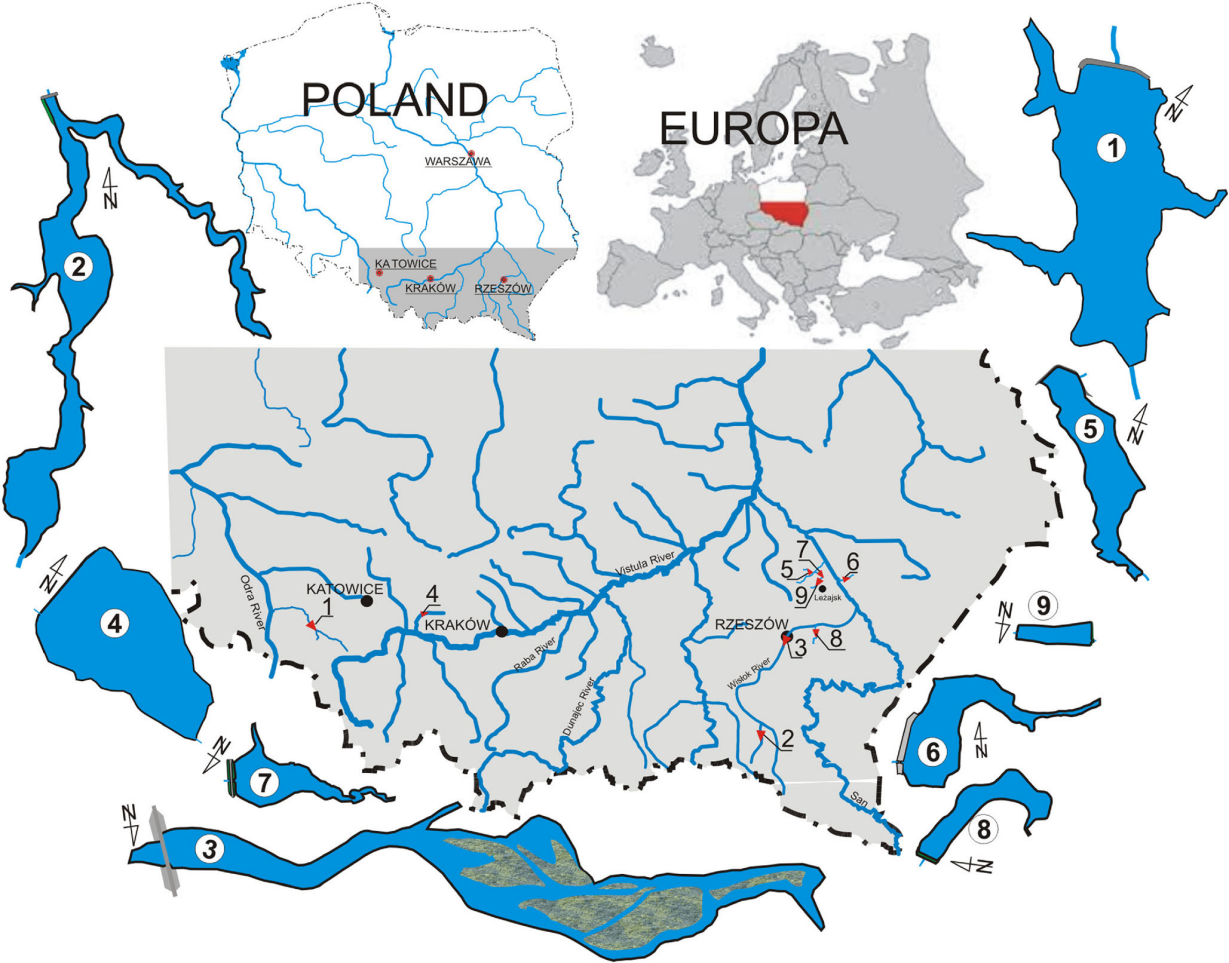
Localization of reservoirs. 1 Rybnik, 2 Besko, 3 Rzeszów, 4 Chechło, 5 Narożniki, 6 Ożanna, 7 Brzóza Królewska, 8 Głuchów, 9 Brzóza Stadnicka

Based on their capacity, the water reservoirs under study can be divided into small and large. Large reservoirs include the Rybnik, Besko and Rzeszów (Table 1, Fig. 1). Their common feature is the role they play in the flood protection system as well as the recreation opportunities they offer, particularly angling, thanks to which they are stocked with fish. The Rybnik reservoir was created as a facility of the conventional power plant’s technological line, and its main purpose is to supply water for cooling the power units (Kostecki 2004; Baran and Tarnawski 2015; Baran et al. 2015; Baran et al. 2016). Water is exchanged and cooled in the reservoir (which puts the reservoir under strong thermal pressure), and flow horizontal circulation is forced. The facility is located in the Silesian region (a heavily industrialized part of the country), within the city of Rybnik; a traffic route runs along the coast, with its culverts (bridges) cutting-off the lateral branches of the reservoir (Koniarz et al. 2014). The loss of capacity of the reservoir caused by deposited terrigenous material does not exceed 10–15%. The Besko reservoir is trough-like, and it was formed as a result of backing up the waters of the Wisłok river by a concrete dam in a narrow valley. The purpose of the reservoir is to enable water intake for public purposes (neighboring cities and villages), for energy purposes, and also to run fish farms (Baran et al. 2011; Tarnawski et al. 2015). An important purpose of this facility is to equalize the base flows and particularly the instream (biological) flow. Due to steep rocky banks of the reservoir, recreation is limited to the backwater zone. Bottom materials are deposited mainly in the backwater zone, making it very shallow. Total loss of capacity does not exceed 10%. The facility built in the city of Rzeszów on the Wisłok River has been subjected to an intensive silting process since the very first years of operation. Currently, the loss of capacity is estimated to be more than 60% (Baran and Tarnawski 2012; Baran et al. 2015; Koniarz et al. 2015a). The reservoir is very shallow, and silts form vast islands. The bowl of the reservoir has been rebuilt (narrowed), and there have been attempts to desilt it, and now, a major repair of the reservoir is planned. The water quality status and the silting ratio made it impossible to draw water from the bowl; the water intake for public water supply was located in the backwater zone. In the barrage, which is also a bridge, there is a small water-powered plant.

**Table 1:** Characteristics of water reservoirs

Division of reservoir	Rivers	Year	Catchment area(km^2^)	Total capacity (thousand m^3^)	Surface of flooding (ha)	Length (km)	Max/mean deep (m)	
Large	1. Rybnik	Ruda and Nacyna	1972	316.78	24,000.00	555.0	7.0	11.0/5.5
	2. Besko	Wisłok	1978	207.0	14,200.00	126.0	5.0	29.0/12.0
	3. Rzeszów	Wisłok	1973	2060.7	1800.00	68.2	6.7	4.0/1.6
Small	4. Chechło	Chechło	1960	42.5	600.00	54.4	1.5	3.8/1.5
	5. Narożniki	Dęba	2001	25.0	283.00	28.0	1.30	3.5/1.0
	6. Ożanna	Złota	1978	136.3	252.00	18.0	0.95	3.5/1.4
Very small	7. Brzóza Królewska	Tarlaka	1985	30.4	48.80	6.13	0.44	3.2/1.4
	8. Głuchów	Graniczna	1995	10.12	22.57	1.5	0.33	2.0/1.0
	9. Brzóza Stadnicka	Tarlaka	*1995*	7.6	10.92	1.15	0.19	1.2/0.8

Other measured and studied facilities can be classified into small and very small reservoirs (Table 1). The Chechło reservoir was built between 1944 and 1945 to supply water to the industry. Currently, the reservoir is used for water retention and for equalizing the outflow during overflow periods. It also has a recreational and tourism function, allowing to do water sports and angling. Built on a small watercourse, it does not silt up fast, but the inflow of biogenes is the reason that the backwater and coastal zones are quickly overgrown with vegetation (Zawisza et al. 2014). The Narożniki reservoir was built on the Dębe River. Its purpose is flood protection for towns, improvement of water-soil conditions and economic development of the surroundings through popularization of water sports and recreation. The measurements taken after 4 years of reservoir operation showed almost 0.6% silting ratio (Baran et al. 2015; Michalec and Wałęga 2015). As a result of a failure of the lateral dam, the reservoir was out of service for 3 years. The Ożanna reservoir has a flood protection function, accumulates firefighting water, counteracts strong bottom erosion and enables recreation. In 1998, renovation works were conducted, and 26,000 m^3^ silt was removed from the bottom of the reservoir (which constituted approx. 10.3% of its capacity). Works carried out in 2010 limited desilting operations to coastal zones. There are two water reservoirs built (cascade system) on the Tarlak stream: the Brzóza Stadnicka reservoir (which is very small and located higher) and the Brzóza Królewska reservoir (larger and located at a distance of approx. 5 km) (Koniarz et al. 2015b). The former is responsible for reduction of bottom erosion at the watercourse bed and for local water retention. The latter also has the flood protection and recreational functions. Measurements taken after 7 years of operation showed a considerable reduction in capacity; the silting ratio was almost 26% (Michalec 2014). The facility in Głuchów is a typical agricultural reservoir created by partitioning the Graniczna watercourse (Koniarz et al. 2015b). Areas surrounding the reservoirs are cultivated fields and grasslands; thus, the main function is to improve water regime in the soil. In 2010, the reservoir was rebuilt, the area of the bowl was increased and inclination of the banks was changed so that the reservoir could be better used by anglers.

### Sampling of bottom sediments

In each of the reservoirs, three bottom sediment collection zones were determined: inlet (backwater), middle and outlet (near the dam). The samples were collected at depths of 0–15 cm using an Ekman sampler. At each sampling location, 5–6 subsamples were collected and thoroughly mixed to obtain a homogeneous sample. All the samples were properly marked, and locations were identified using a global positioning system (GPS). A total of 27 samples were stored at − 20 °C until ready for further analysis.

### Chemical analyses

#### Basic properties of sediments

All of the sediment samples were air-dried in the laboratory. Physicochemical properties of the sediment samples (grain size fractions, content of organic matter carbon and total nitrogen) were analyzed and shown in Table 2. The organic matter carbon (C_OM_) content was determined by loss-on-ignition for 8 h at 450 °C. The aerometric method was used to analyze the grain size fractions. The content of total nitrogen (TN) in sediments was determined by Kjeldahl methods.

**Table 2:** Statistical evaluation of basic properties of the sediment

Bottom sediment		Grain size fraction (%)			C_OM_ %	TN %	C/N
		Sand	Silt	Clay			
Rybnik	Mean	–	–	–	24.67	0.92	27
	SD	–	–	–	5.43	0.01	6
	Min	34	22	23	18.86	0.91	21
	*Max*	54	43	25	29.62	0.92	32
	*CV%*	–	–	–	22	1	22
Besko	Mean	–	–	–	1.82	0.14	13
	SD	–	–	–	0.61	0.01	5
	Min	5	11	84	1.14	0.13	8
	*Max*	7	15	90	2.32	0.15	18
	*CV%*	–	–	–	34	8	39
Rzeszów	Mean	–	–	–	2.5	0.23	11
	SD	–	–	–	0.5	0.06	1
	Min	7	35	48	1.93	0.17	10
	*Max*	9	45	57	2.89	0.29	11
	*CV%*	–	–	–	20	25	7
Chechło	Mean	–	–	–	9.15	110	20
	SD	–	–	–	7.78	95	15
	Min	68	12	8	0.99	10	2
	*Max*	80	15	17	16.47	200	30
	*CV%*	–	–	–	85	87	77
Narożniki	Mean	–	–	–	0.89	0.06	14
	SD	–	–	–	0.58	0.04	2
	Min	26	1	5	0.22	0.02	12
	*Max*	89	46	28	1.27	0.08	17
	*CV%*	–	–	–	65	60	16
Ożanna	Mean	–	–	–	3.08	0.31	11
	SD	–	–	–	1.12	0.14	2
	Min	47	8	10	1.93	0.15	9
	*Max*	82	23	30	4.15	0.41	13
	*CV%*	–	–	–	36	46	21
Brzóza Królewska	Mean	–	–	–	2.55	0.2	11
	SD	–	–	–	3.3	0.23	2
	Min	43	6	4	0.16	0.02	10
	*Max*	90	24	33	6.31	0.46	14
	*CV%*	–	–	–	129	119	17
Głuchów	Mean	–	–	–	1.42	0.14	9
	SD	–	–	–	1.46	0.11	3
	Min	15	43	28	0.36	0.06	6
	*Max*	20	52	41	3.08	0.26	12
	*CV%*	–	–	–	103	81	34
Brzóza Stadnicka	Mean	–	–	–	2.93	0.21	14
	SD	–	–	–	1.25	0.11	1
	Min	49	5	5	1.62	0.11	13
	*Max*	90	27	24	4.12	0.32	15
	*CV%*	–	–	–	43	50	9
*Mean*	–	–	–		5.45	0.29	15
*Minimum*		5	1	4	0.16	0.02	2
*Maximum*		90	52	84	29.62	0.92	32

#### Extraction and analysis of PAHs

The samples were dried in darkness at room temperature for approximately 48 to 72 h. For PAH determination, 5 g of air-dried sediment sample was extracted in an ultrasonic bath with 10 mL of acetonitrile for 60 min. The sample was put aside for 10 min, and the solid fraction was removed by filtration. The extracts were purified by column chromatography using CHROMAFIX 400-SA cartridge solid phase extraction (SPE). The fraction of 11 PAHs was isolated from the sediment samples by solid phase extraction (SPE). First, the CHROMAFIX 400-SA cartridges were conditioned with 3 mL of methanol. One milliliter of the sample was passed through a prepared cartridge, and the PAHs were eluted with methanol (2 × 1 mL). Then, the extracts were concentrated in a gentle stream of high-purity nitrogen to a volume of 1 mL and solvent-exchanged into that of dichloromethane (Sample preparation SPE Macherey-Nagel n.d.). The concentration of 11 PAHs (naphthalene [NAP], acenaphthylene [ACL], acenaphthene [ACN], fluorene [FLN], phenanthrene [PHE], anthracene [ANT], fluoranthene [FLT], pyrene [PYR], benzo(a)anthracene [BAA], chrysene [CHR], benzo(a)pyrene [BAP]) was determined by gas chromatography-mass spectrometry on a Varian GC/MS/MS 4000 apparatus equipped with ion trap. Resolution of PAH compounds has been achieved using a FactorFour VF-5MS capillary column (length 30 m, internal diameter 0.25 mm, film thickness 0.25 μm). The carrier gas was helium at a flow rate of 1 mL min^−1^. The injector temperature and transfer line temperature were set at 325 °C. The GC oven temperature was programmed as follows: 40 °C for 2 min, followed by a 30 °C min^−1^ ramp to 250 °C and then with ramp 10 °C min^−1^ to final temperature 320 °C (2.5 min). Total time of the analysis was 18.5 min. The mass spectrometer operated at electron energy of 70 eV, with an ion source temperature of 150 °C. MS detector operated in SCAN mode, and the ranges of mass scanning were between m/z 70 and 300. The identification of PAHs in the sediment samples was achieved by matching the retention time and mass spectra for each PAH with those determined for an external standard. Restek—“610 PAH Calibration Mix B” standard was used (16 PAHs in methylene chloride:methanol (1:1, v:v, 100–2000 μg/mL)) (Jasiewicz et al. 2007). The recoveries were 98.5% for NAP, 90.6% for ACL, 92.2%, for ACN, 86.4% for FLN, 89% for PHE, 88% for ANT, 90% for FLT, 85% for PYR, 84% for BAA, 80% for CHR and 83% for BAP. In addition, limit of detection (LOD) was 2 μg kg^−1^ DM, and limit of quantification (LOQ) was 6 μg kg^−1^ DM.

### Statistical analysis

The results were expressed as mean ± standard deviation (SD), minimum and maximum values and coefficient of variation (CV%) of triplicate determinations. Pearson’s correlation matrix and principal component analysis (PCA) were used to explore the possible relationships between PAHs in sediments. The differences between means were also determined using one-factor analysis ANOVA and LSD test at significance level of 0.05. In order to meet the principles of the analysis of variance (additivity, homogeneity of variance and normality of distribution), the data were subjected to logarithmic transformation prior to the analysis. All statistical analyses were performed using Microsoft Office Excel and STATISTICA 12.5 software.

## Results and discussion

### Sediment characteristics

The grain size fraction, concentration of C_OM_, TN and C_OM_/TN ratio are provided in Table 2. The sediments showed a high diversity of grain size fraction distribution. The bottom material from the Rybnik reservoir contains 34 to 54% sand, 22 to 43% silt and 23 to 25% clay. The clay fraction was dominant in the bottom sediments collected from the Besko (84–90%) and Rzeszów (48–57%) reservoirs. Generally, in the bottom sediments collected from the Narożniki, Brzóza Królewska and Brzóza Stadnicka, Ożanna and Chechło reservoirs, the sand fraction was dominant. Silt was the dominant grain size fraction in sediments from Głuchów (Table 2). Organic carbon content in surface sediments depends on a series of factors such as sedimentary characteristics, rate of microbial degradation, column water productivity and terrestrial inputs (Sampei and Matsumoto 2001; Burone et al. 2003). The C_OM_ content in the samples varied from 0.16 to 29.62%. Sediments with the highest C_OM_ content were found in the Rybnik reservoir. The lowest mean C_OM_ content was observed in the bottom sediment from the Narożniki reservoir. TN content in the bottom sediments ranged between 0.02 (Narożniki) and 0.92% (Rybnik). The C/N ratio in aquatic systems is governed by the mixing of terrestrial and autochthonous organic carbon. Higher plants have a lower nitrogen content and thus a higher C:N ratio. High C/N ratios (15 or higher) in sediments indicate contribution of terrigenous organic carbon. Phytoplankton and zooplankton are rich in nitrogen compounds, and low C:N ratios (5 to 9) of sediments indicate a dominance of autochthonous organic matter (Burone et al. 2003). The C/N ratio in sediments ranged from 2 to 32 (Table 2). The highest C/N ratios were found in sediments from the Rybnik and Chechło reservoirs. This suggests that the deposition of terrigenous material is dominant in both reservoirs. Generally, most sediment samples had C/N ratios between 9 and 18, which indicates a weaker terrigenous influence. The lowest C/N ratios were found in sediments from the Głuchów reservoir (Table 2).

### PAH concentration and sources

**Fig. 2:**
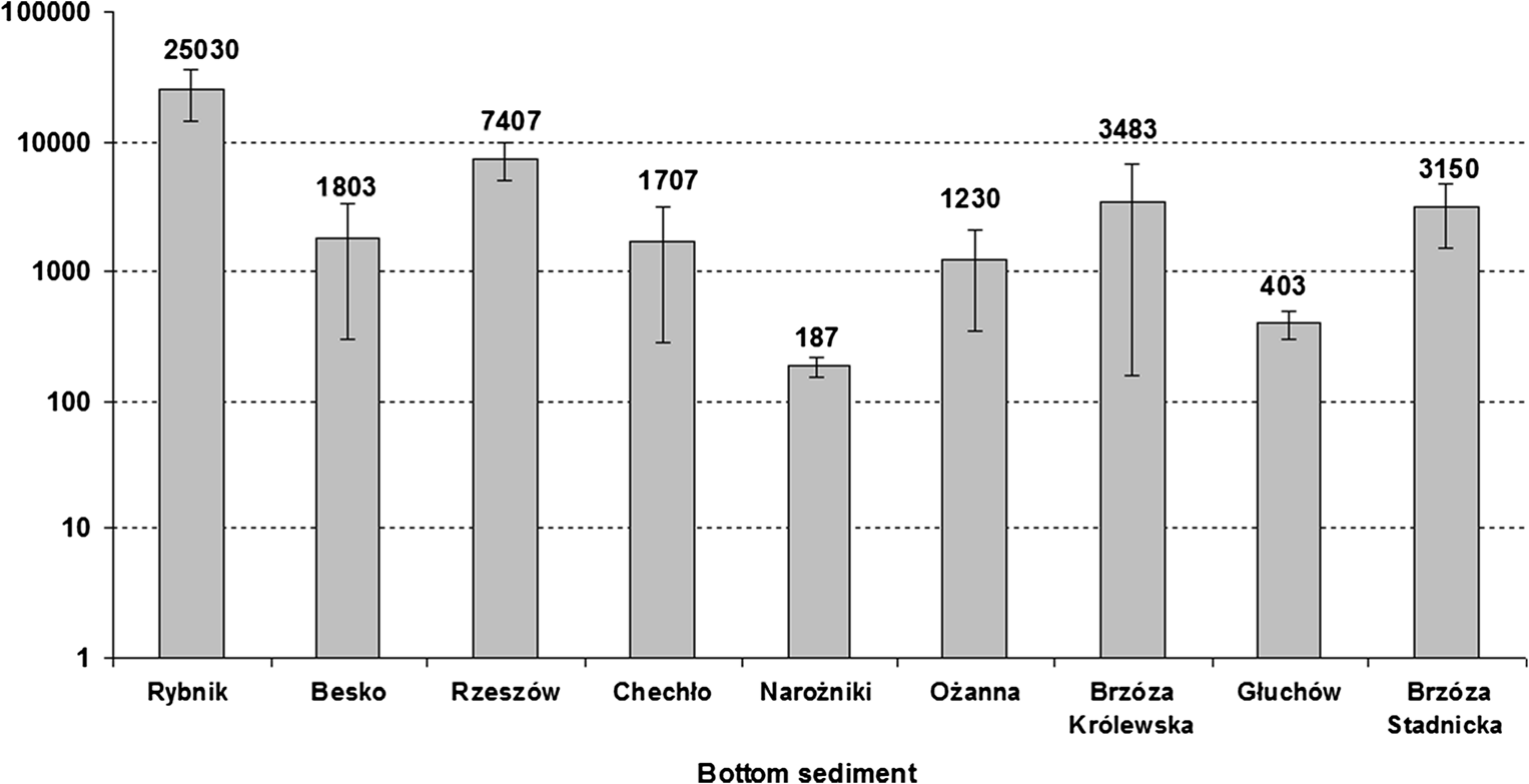
Total PAH content in the bottom sediment (μg kg^−1^ DM)

The concentrations of individual PAHs and total PAH concentrations in surface sediment are given in Tables 3–4 and Fig. 2. The PAH concentrations in sediments varied widely among the nine water reservoirs. However, statistically significant differences were detected only for some hydrocarbons found in bottom sediments of the Rybnik, Chechło and Rzeszów reservoirs. From the PAHs, FLT was represented most strongly—its concentrations in the sediment samples from the reservoir have average values of 1255 μg kg^−1^ DM. The second in line was PYR, with the average level being 958 μg kg^−1^ DM. These compounds also had the highest maximum values: 9010 (FLT) and 6980 μg kg^−1^ DM (PYR), respectively. These samples also had the highest variability in terms of the average value. Benzo(a)pyrene also had a quite high content (with mean value of 798 μg kg^−1^ DM) in the sediments, whereas relatively low maximum values for this compound were detected. The lowest mean concentrations of PAHs were found for anthracene, fluorene, acenaphthene and acenaphthylene. These compounds were also the least diversified. The total concentration of PAHs in surface sediments ranged from 150 to 33,900 μg kg^−1^ DM, with a mean concentration of 4933 μg kg^−1^ DM (Table 4). The highest total PAH concentrations were recorded in sediments from the Rybnik (mean 25,030 μg kg^−1^ DM). The lowest total PAH concentrations, ranging from 150 to 210 μg kg^−1^ DM, were observed in sediments from the Narożniki reservoirs. The total PAH concentration in the bottom sediments is arranged in the following order: Rybnik > Rzeszów > Brzóza Królewska > Brzóza Stadnicka > Besko > Chechło > Ożanna > Głuchów > Narożniki (Fig. 2). The main reasons for relatively high PAH concentrations in the bottom sediments of the Rybnik and Rzeszów reservoirs are probably their proximity to urbanized areas. Generally, the source of PAHs in the bottom sediment of the Rzeszów and Rybnik reservoirs is incomplete combustion process connected with industry, transport and low emissions from individual sources of heating during the winter period. PAHs are preferentially associated with particulate matter, so atmospheric fallout is a principle route of contamination. Especially PAHs with four or more aromatic rings are found predominantly on particulates (usually as small as < 2.5 μm) (Maliszewska-Kordybach et al. 2009; Abdel-Shafy and Mansour 2016). The important point sources of PAHs in the catchment area of the Rybnik reservoir are also sewage treatment plants. Moreover, high content of organic matter (Rybnik reservoir) and clay fraction (Rzeszów reservoir) of the soil and sediment system is the most important factor in the sorption of polycyclic aromatic hydrocarbons and thus their accumulation in the environment (Lahlou and Ortega-Calvo 1999; Müller et al. 2007; Crampon et al. 2014; Junttila et al. 2015; Abdel-Shafy and Mansour 2016). Sediments from the Chechło, Brzóza Stadnicka and Brzóza Królewska reservoirs also had relatively a high C_OM_ content and high C/N ratio values (Table 2). This may indicate that high concentrations of PAHs in the bottom sediments of the previously mentioned reservoirs are related with uncontrolled sanitary sewer effluents and roadway runoff. Moreover, sediments from the Brzóza Stadnicka reservoir and the Brzóza Królewska reservoir were also similar to the mean total PAH concentrations. This situation was probably caused by the fact that these reservoirs are located in the same river basin. Brzóza Stadnicka enclosed in the basin of Brzóza Królewska acts as an initial reservoir, capturing a portion of pollutants flowing in the Tarlak river (Koniarz et al. 2015b). Moreover, the lowest PAH concentrations in the sediments from the Narożniki and Głuchów reservoirs could be associated with a low C_OM_ content and dominant sandy fraction. Moreover, both reservoirs are located in rural areas, and these areas were characterized by low anthropopressure related to urbanization, industry and transport (Madeyski et al. 2008, Baran et al. 2015). The highest PAH concentration in sediments from the Rybnik, Głuchów, Brzóza Królewska, Brzóza Stadnicka, Besko and Chechło reservoirs was found at station 3 near the dam, while in sediments from the Rzeszów and Narożniki reservoirs—at station 1 (inlet, backwater station). BAP was the major compound, accounting for approximately 30% of total PAHs in sediments from the Besko, Brzóza Stadnicka and Rzeszów reservoirs (Table 4). Among all 11 PAHs, FLT was the predominant species and accounted approximately for 29 (Rybnik reservoir), 40 (Narożniki reservoir), 30 (Ożanna reservoir) and 36% (Brzóza Królewska reservoir) of total PAHs. In addition, FLN (38% of total PAHs) was prevalent in samples from the Głuchów reservoir, and NAP (32% of total PAHs) in the sediments from Chechło (Tables 3, 4). ∑PAH pollution levels are classified into four categories (μg ∑PAH kg^−1^ DM): low (0–100), moderate (100–1000), high (1000–5000) and very high (>5000) (Baumard et al. 1999). Generally, the studied sediments are contaminated with PAHs at very high (Rybnik, Rzeszów reservoirs), high (Brzóza Stadnicka, Brzóza Królewska, Chechło, Ożanna, Besko reservoirs) and moderate (Głuchów, Narożniki reservoirs) degrees.

**Table 3:** Concentration of PAHs (μg kg^−1^ DM) in bottom sediments

Bottom sediment	NAP	ACL	ACN	FLN	PHE	ANT	FLT	PYR	
Rybnik	Mean^5^	633c^2^	nd^1^	nd	257b	4527b	413c	7140c	5237c
	SD^3^	337	nd	nd	78	2155	197	2499	1889
	Min	250	nd	nd	170	2090	280	4300	3230
	*Max*	880	nd	nd	320	6180	640	9000	6980
	*CV%* ^4^	53	nd	nd	30	48	48	35	36
Besko	Mean	47a	nd	nd	nd	160a	nd	277ab	247ab
	SD	12	nd	nd	nd	35	nd	289	247
	Min	40	nd	nd	nd	140	nd	90	80
	*Max*	60	nd	nd	nd	200	nd	610	530
	*CV%*	25	nd	nd	nd	22	nd	105	100
Rzeszów	Mean	47a	nd	nd	nd	460a	73ab	1710b	1417b
	SD	6	nd	nd	nd	104	29	582	435
	Min	40	nd	nd	nd	340	40	1100	970
	*Max*	50	nd	nd	nd	520	90	2260	1840
	*CV%*	12	nd	nd	nd	23	39	34	31
Chechło	Mean	350b	33	23	63a	110a	183b	147a	67a
	SD	182	58	32	101	95	166	127	60
	Min	140	nd	nd	nd	10	30	10	10
	*Max*	460	100	60	180	200	360	260	130
	*CV%*	52	173	138	160	87	91	86	90
Narożniki	Mean	nd	nd	nd	nd	70a	nd	73a	40a
	SD	nd	nd	nd	nd	50	nd	12	35
	Min	nd	nd	nd	nd	30	nd	60	0
	*Max*	nd	nd	nd	nd	87	nd	80	60
	*CV%*	nd	nd	nd	nd	71	nd	16	87
Ożanna	Mean	13a	nd	nd	nd	90a	nd	277ab	210a
	SD	23	nd	nd	nd	35	nd	127	125
	Min	nd	nd	nd	nd	70	nd	140	80
	*Max*	40	nd	nd	nd	130	nd	390	330
	*CV%*	173	nd	nd	nd	38	nd	46	60
Brzóza Królewska	Mean	nd	nd	nd	nd	100a	nd	840ab	707ab
	SD	nd	nd	nd	nd	45	nd	750	645
	Min	nd	nd	nd	nd	150	nd	90	60
	*Max*	nd	nd	nd	nd	200	nd	1590	1350
	*CV%*	nd	nd	nd	nd	45	nd	89	91
Głuchów	Mean	40a	nd	nd	nd	nd	nd	157a	80a
	SD	13	nd	nd	nd	nd	nd	45	35
	Min	35	nd	nd	nd	nd	nd	110	60
	*Max*	50	nd	nd	nd	nd	nd	200	120
	*CV%*	33	nd	nd	nd	nd	nd	29	43
Brzóza Stadnicka	Mean	12a	nd	nd	nd	143a	nd	710ab	617ab
	SD	13	nd	nd	nd	95	nd	407	349
	Min	nd	nd	nd	nd	70	nd	460	410
	*Max*	40	nd	nd	nd	250	nd	1180	1020
	*CV%*	109	nd	nd	nd	66	nd	57	57
*Mean*		*127*	*nd*	*nd*	*36*	*646*	*74*	*1255*	*958*
*Minimum*		*40*	*100*	*10*	*nd*	*10*	*30*	*10*	*10*
*Maximum*		*880*	*100*	*60*	*320*	*6180*	*640*	*9010*	*6980*

^1^nd—not detected

^2^Means followed by the same letters in line did not differ significantly at α ≤ 0.05 according to the LSD test

^3^SD—standard deviation

^4^CV%—coefficient of variation

^5^Mean value for the three zones in the reservoir

**Table 4:** Concentration of PAHs (μg kg^−1^ DM) in bottom sediments and diagnostic ratios of PAHs in bottom sediment

Bottom sediment	BAA	CHR	BAP	LWM	HWM	LWM/HWM	FLU/(FLU + PYR)	
Rybnik	Mean^5^	1747c^2^	3017c	2053b	5837	19,193	0.30	0.58
	SD^3^	504	1218	2040	2617	8009	0.33	0.02
	Min	1210	1610	0	2900	10,360	0.28	0.56
	*Max*	2210	3730	4080	7920	25,980	0.31	0.60
	*CV%* ^4^	29	40	99	45	42	107	4
Besko	Mean	193a	180a	693ab	213	1590	0.42	0.53
	SD	168	312	603	44	1462	0.56	0.03
	Min	nd	nd	nd	180	170	0.08	0.50
	*Max*	300	540	1100	260	3090	1.07	0.55
	*CV%*	87	173	87	21	92	133	6
Rzeszów	Mean	657b	1063b	1977b	583	6822	0.09	0.54
	SD	216	325	690	133	2205	0.01	0.01
	Min	410	730	1180	430	4400	0.08	0.53
	*Max*	810	1380	2400	660	8700	0.1	0.55
	*CV%*	33	31	35	23	32	11	2
Chechło	Mean	177a	190ab	370a	760	948	1.24	0.68
	SD	170	134	291	585	842	0.83	0.07
	Min	10	10	50	180	80	0.75	0.62
	*Max*	350	390	620	1350	1760	2.19	0.75
	*CV%*	96	71	79	77	89	67	10
Narożniki	Mean	nd^1^	nd	nd	70	117	0.67	0.69
	SD	nd	nd	nd	0	31	0.22	0.27
	Min	nd	nd	nd	70	80	0.51	0.5
	*Max*	nd	nd	nd	70	140	0.92	1
	*CV%*	nd	nd	nd	0	27	33	39
Ożanna	Mean	77a	97a	467a	103	1127	0.16	0.59
	SD	75	106	412	58	834	0.15	0.05
	Min	nd	nd	nd	70	220	0.06	0.54
	*Max*	150	210	780	170	1860	0.33	0.64
	*CV%*	98	110	88	56	74	94	8
Brzóza Królewska	Mean	307ab	693ab	840ab	100	3383	0.02	0.56
	SD	305	589	460	100	3225	0.02	0.03
	Min	nd	nd	nd	0	160	0	0.54
	*Max*	610	1390	1680	200	6610	0.03	0.6
	*CV%*	99	85	55	100	95	100	5
Głuchów	Mean	nd	nd	nd	193	210	1	0.61
	SD	nd	nd	nd	45	78	0.35	0.03
	Min	nd	nd	nd	150	160	0.68	0.58
	*Max*	nd	nd	nd	240	300	1.38	0.64
	*CV%*	nd	nd	nd	23	37	35	5
Brzóza Stadnicka	Mean	290ab	593ab	780ab	157	2994	0.05	0.54
	SD	148	371	246	117	1519	0.02	0.01
	Min	190	350	600	70	2090	0.03	0.53
	*Max*	460	1020	1060	290	4740	0.06	0.54
	*CV%*	51	62	32	75	51	40	2
*Mean*		*383*	*648*	*798*	891	4043	0.44	0.59
*Minimum*		*10*	*10*	*50*	0	80	0	0.5
*Maximum*		*2210*	*3740*	*4080*	7920	25,980	2.19	1

^1^nd—not detected

^2^Means followed by the same letters in line did not differ significantly at α ≤ 0.05 according to the LSD test

^3^SD—standard deviation

^4^CV%—coefficient of variation

^5^Mean value for the three zones in the reservoir

**Fig. 3:**
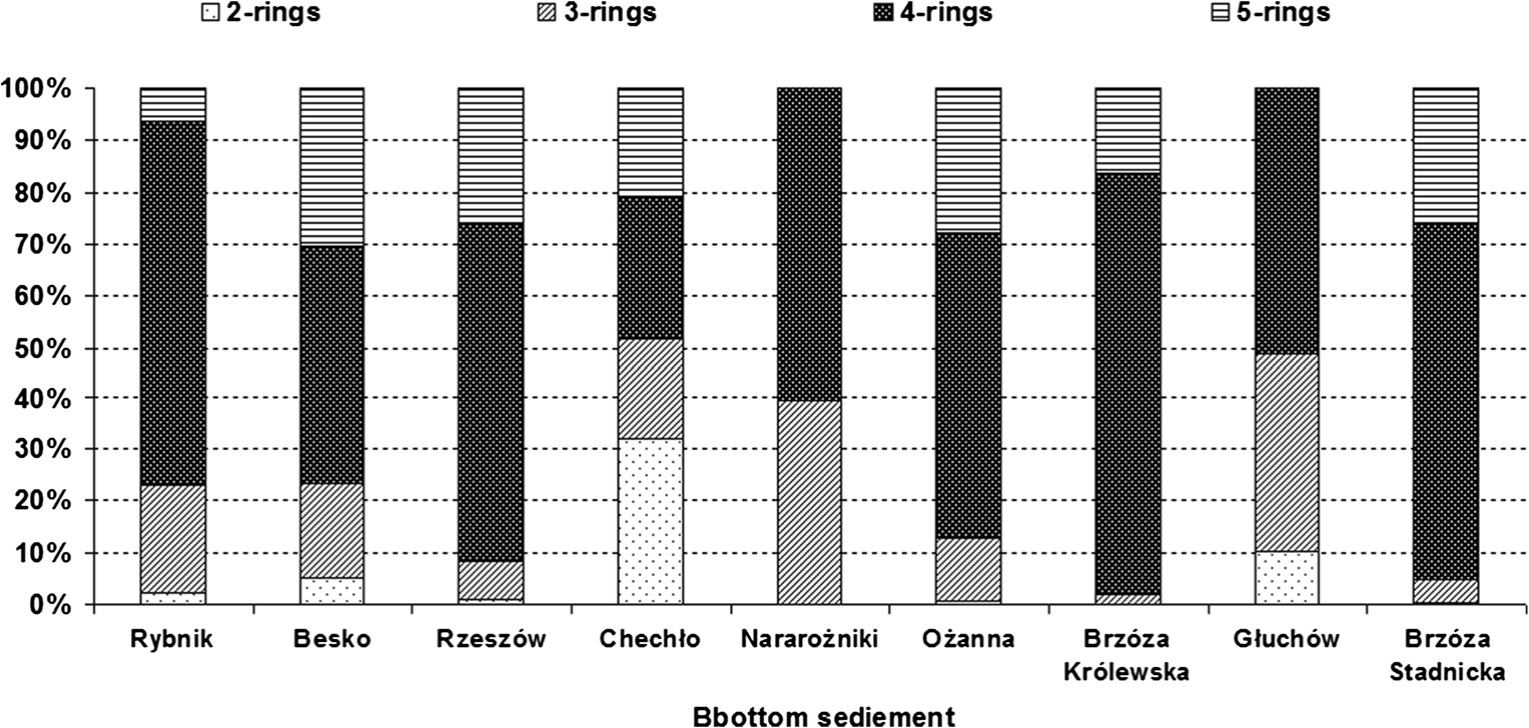
Distribution of 2-, 3-, 4- and 5-ring PAHs in the bottom sediments

Based on characteristics in PAH composition and distribution pattern, the sources of anthropogenic PAHs can be distinguished by ratios of individual PAH compounds (Dahle et al. 2003; Yan et al. 2009; Wang et al. 2013). Generally, petrogenic PAHs are characterized by the predominance of 2- and 3-ring PAHs, while pyrogenic PAHs are characterized by high proportion of above 4-ring PAHs (Wang et al. 2013). Moreover, LMW/HMW ratio < 1 (low molecular weight parent PAHs (2–3 ring PAHs)/high molecular weight parent PAHs (4–6 ring PAHs except perylene) suggests pollution of pyrolytic origin (Yan et al. 2009; Hiller et al. 2009; Tavakoly Sany et al. 2014). A low LMW/HMW ratio is also attributed to high resistances of the high molecular weight PAHs to microbial degradation (Tavakoly Sany et al. 2014). Other authors observed that a low ratio could be caused by high solubility and volatility of low molecular weight PAHs (Chandru et al. 2008; Elias et al. 2009). The composition pattern of PAHs by ring size in sediments is shown in Fig. 3. Generally, 4-ring PAHs were predominant in the sediment samples. Only in the sediments from the Chechło reservoir 2-ring PAHs and in the sediment from the Besko reservoir 5-ring PAHs were most abundant. Two-ring compounds ranged from undetected amounts (Narozniki, Brzóza Stadnicka, Brzóza Królewska reservoirs) to 32% (Chechło reservoir); 3-ring compounds ranged from 2 (Brzóza Królewska reservoir) to 39% (Narożniki, Głuchów reservoirs); 4-ring compounds ranged from 27 (Besko reservoir) to 82% (Brzóza Królewska); and 5-ring ranged from undetected amounts (Głuchów, Narożniki reservoirs) to 30% (Besko reservoir) of the total PAH concentration (Fig. 3). The high molecular weight PAHs in the present study contributed between 48 (Chechło reservoir) and 98% (Brzóza Królewska reservoir) (mean 76%) (Fig. 3, Table 4) to the overall concentration of PAHs, which implies domination of pyrogenic sources. The low molecular weight PAHs in the present study contributed between 2 (Brzóza Królewska reservoir) and 51% (Chechło reservoir). As shown in Table 4, LMW/HMW ratios were generally below 1, suggesting also a pyrolytic origin. The high concentration of 2- and 3-ring PAHs was found in sediments from the Chechło reservoir (station 3) and the Głuchów reservoir, which suggests that the petrogenic source is important (Fig. 3, Tables 3, 4). FLU/(FLU + PYR) ratio can be also used as an indicator of PAH origin (Table 4). FLU/(FLU + PYR) ratio < 0.4 is attributed to petrogenic source, and ratio > 0.5 is suggested to that of wood and coal combustion, while between 0.4 and 0.5 is characteristic of petroleum combustion (Yan et al. 2009; Hiller et al. 2009; Tavakoly Sany et al. 2014). In our study, FLU/(FLU + PYR) ratios were higher than 0.5, which indicates that pyrolytic inputs (kerosene, grass, wood and most coal combustion) are the major source of PAHs in the reservoir sediments.

### Ecological risk assessment to benthic fauna

We assessed a potential hazard to the organisms connected with PAHs in the sediments using numerical sediment quality guidelines (SQGs) of the numerical indices: threshold effect concentration (TEC) and probable effect concentration (PEC) (MacDonald et al. 2000). PAH concentrations at each station were compared with the consensus-based sediment quality guideline values referred to as TEC and PEC (Table 5). In the risk characterization step, the hazard quotient (HQ) was calculated based on the ratio between the concentration of each individual PAH and the concentration to its corresponding PEC value (Khairy et al. 2009; Tavakoly Sany et al. 2014). When HQ > 1, frequent adverse ecological effects are expected (Khairy et al. 2009). In the study, the mean PEC quotient (PECq) for nine PAHs was determined. The mean PECq is the average of the ratios between the PAH concentration in sediments and the related PEC value. When mean PECqs were < 0.5, sediment samples were predicted to be non-toxic, indicating low potential toxicity to the benthic fauna. When mean PECqs > 0.5, sediment samples were toxic, indicating a high potential risk to the benthic fauna (MacDonald et al. 2000). The results obtained from the ecological risk assessment of PAHs in sediments are summarized in Table 5. Mean PECqs of PAHs ranged from 0.01 to 2.01. The highest mean values of PECqs were found in sediments from the Rybnik reservoir. The lowest mean values of PECqs were characteristic for sediments from the Narożniki and Głuchów reservoirs. Generally, the risk assessment revealed that total PAHs are likely to cause high potential toxicity to biological communities (PECqs > 0.5) in sediments from the Rybnik and Rzeszów reservoirs. Additionally, low potential toxicity to the benthic fauna (PECqs < 0.5) was observed for sediments from the Głuchów, Narożniki, Besko, Ożanna, Chechło, Brzóza Stadnicka and Brzóza Królewska reservoirs. Other authors divided PECqs into four categories: non-adverse effect (PECq < 0.1), slightly adverse effect (0.1 < PECq < 0.5), moderate effect (0.5 < PECq < 1.0) and strong effect (PECq > 1.5) (Ingersoll et al. 2001; Tavakoly Sany et al. 2014). In these ranges, non-adverse effects on the benthic fauna were shown for sediments from the Głuchów, Narozniki and Ożanna reservoirs, while slightly adverse effects were found for sediments from the Brzóza Królewska, Besko, Brzóza Stadnicka and Chechło reservoirs. The other sediments showed moderate (Rzeszów reservoirs) and strong effect (Rybnik reservoir) on biological communities (Table 5). Based on a comparison with the sediment guidelines, individual PAHs such as the NAP, PHE, FLT, PYR, BAA, CHR and BAP in sediments from the Rybnik reservoir and BAP in sediments from the Rzeszów reservoir indicated a higher possibility of occurrence of an adverse ecological effect (Table 5). The sediment samples were predicted to be non-toxic when the measured concentrations of individual PAHs were lower than the corresponding TECs (Macdonald et al. 2000). According to the results of the ecological risk assessment, it can be concluded that none of the PAHs are contaminants of potential concern only in sediments from Głuchów and Narożniki.

**Table 5:** Hazard quotients calculated for PAHs in the sediment of different reservoirs

Bottom sediments	NAP	FLN	PHE	ANT	FLT	PYR	BAA	CHR	BAP	PECq^1^
Rybnik	1.13	0.48	3.87	0.49	3.2	3.45	1.67	2.34	1.41	2.01
Besko	0.08	0	0.14	0	0.13	0.16	0.19	0.14	0.48	0.14
Głuchów	0.07	0	0.13	0	0.06	0.05	0	0	0	0.04
Narozniki	0	0	0.06	0	0.04	0.03	0	0	0	0.01
Ożanna	0.02	0	0.08	0	0.12	0.14	0.07	0.07	0.32	0.09
Brzóza Królewska	0	0	0.08	0	0.38	0.46	0.29	0.54	0.58	0.26
Brzóza Stadnicka	0.02	0	0.12	0	0.32	0.41	0.28	0.46	0.54	0.24
Rzeszów	0.08	0	0.4	0.08	0.77	0.93	0.63	0.83	1.36	0.57
Chechło	0.62	0.12	0.09	0.21	0.07	0.04	0.17	0.15	0.25	0.19
*Mean*	*0.22*	*0.07*	*0.55*	*0.09*	*0.57*	*0.63*	*0.37*	*0.5*	*0.55*	*0.39*
*Minimum*	*0*	*0*	*0.06*	*0*	*0.04*	*0.03*	*0*	*0*	*0*	*0.01*
*Maximum*	*1.13*	*0.48*	*3.87*	*0.49*	*3.2*	*3.45*	*1.67*	*2.34*	*1.41*	*2.01*
^*a*^ *TEC μg kg* ^*−1*^	*176*	*77.4*	*204*	*57.2*	*423*	*195*	*108*	*166*	*150*	–
^*a*^ *PEC μg kg* ^*−1*^	*561*	*536*	*1170*	*845*	*2230*	*1520*	*1050*	*1290*	*1450*	–

^a^Macdonald et al. (2000)

^1^PECq—probable effect concentration quotient

### Relationship between PAH concentrations and sediment properties

Table 6 shows correlation coefficients between PAH concentrations in the bottom sediments and selected parameters of the sediments. The hydrophobicity of the solute and total organic matter is an important parameter governing the distribution and adsorption of PAHs in the bottom sediments (Dahle et al. 2003; Hiller et al. 2009; Costa et al. 2011; Arndt et al. 2013; Wang et al. 2013). Due to the high hydrophobicity, PAHs have a high affinity to organic matter. In addition, not only the overall content of organic matter in sediments, but organic matter composition, is important in sorption processes. The highest sorption capacities for PAHs are characterized by humins, less humic acids and the weakest fulvic acids. Other research studies point to the key role of dissolved organic carbon (SOC) and black carbon (BC) in sorption and desorption processes of PAHs e.g. high molecular weight PAHs have high affinity to BC (Klimkowicz-Pawlas et al. 2016). Other important parameters that affect the retention and release of hydrophobic organic chemicals are temperature, conductivity, pH and clay content (Hiller et al. 2009; Junttila et al. 2015). Smectite, illites and kaolinite are the three common clay minerals which have the greatest impact on sorption/desorption because of their high surface area and CEC as well as their surface reactivity (Junttila et al. 2015). Small-sized particles have large specific areas and high adsorption capacity of organic compounds (Dahle et al. 2003; Wu et al. 2016). Many authors observed that organic carbon and sediment grain size are positively or negatively correlated with organic compounds, especially if the absorbents and adsorbents are of the same source (Lubecki and Kowalewska 2010; Ali et al. 2014). McElroy et al. (1989) and Junttila et al. (2015) found that PAHs have a strong affinity for organic matter and therefore tend to accumulate in fine-grained sediments. The correlation between PAH and clay content implies that PAHs are trapped in the fine-grained sediments (Junttila et al. 2015). The nonlinear correlation between organic carbon and PAH concentrations in sediments suggests that the PAHs were recently generated and therefore were yet to fully partition into organic matter in sediments (Sojinu et al. 2010). In our study, correlation coefficients between PAHs and C_OM_ content were positive, statistically significant and mostly above 0.7, except for BAP (insignificant correlation). The same relationships were found for total nitrogen content and C/N ratio (Table 6). High statistically significant correlations between PAH concentrations and C_OM_ in sediments (*r* = 0.71 for ∑PAHs and C_OM_) demonstrate that these pollutants have a strong affinity for organic matter in the studied sediments. Generally, PAH concentration showed an insignificant correlation with grain size. Only for BAP concentration, found significant positively correlation with slightest fraction (Table 6). This shows that BAP in sediments is associated mainly with clay fraction. Many authors also found a strong relationship between PAH concentrations and organic matter content or clay fraction in bottom sediments (Oros and Rosa 2004; Khairy et al. 2009; Lubecki and Kowalewska 2010; Junttila et al. 2015). However, in some regions, an insignificant or weak significant correlation between PAH concentrations and organic carbon or clay fraction in sediments was observed (Cortazar et al. 2008; Sojinu et al. 2010; Tavakoly Sany et al. 2014). This may suggest that the majority of the target compounds were not sorbed on the external surface of the sediment particles, and other factors should be considered (Wu et al. 2016). Wu et al. (2016) found that distribution of organic compounds in the bottom sediments can be influenced by distribution and chemistry of anthropogenic debris. Concentrations of individual PAHs were significantly positively correlated with one another (Table 6). However, correlation coefficients between BAP and other PAHs were the lowest. There is a correlation between all 4- and 6-ring groups of PAHs, which points to a common source of origin, namely combustion processes. The study also showed a significant correlation between BAP and ∑PAH. Some authors found BAP as a potential marker in the study of PAH pollutants and is the only compound for which toxicological data allow derivation of carcinogenic potency factor. This compound could be chosen as an indicator of total PAH concentration and to assess the sediment contamination level (Magi et al. 2002; Wang et al. 2016). Particularly, BAP can be used as an indicator of some combustion-derived PAHs since its concentration in petroleum is usually negligible (Magi et al. 2002; Sojinu et al. 2010).

**Table 6:** Correlation coefficients of PAH concentrations in the sediments with sediment properties (*n* = 54)

Parameter	NAP	ACL	ACN	FLN	PHE	ANT	FLT	PYR	BAA	CHR	BAP
ACL	0.27										
ACN	0.32	0.99									
FLN	0.78*	0.32	0.31								
PHE	0.85*	− 0.05	− 0.07	0.81*							
ANT	0.88*	0.37	0.39	0.93*	0.84*						
FLT	0.81*	− 0.09	− 0.10	0.80*	0.98*	0.82*					
PYR	0.78*	− 0.10	− 0.11	0.80*	0.97*	0.82*	0.98*				
BAA	0.78*	− 0.01	− 0.02	0.81*	0.93*	0.84*	0.97*	0.98*			
CHR	0.77*	− 0.05	− 0.07	0.73*	0.92*	0.77*	0.97*	0.98*	0.98*		
BAP	0.52*	− 0.04	− 0.05	0.44	0.68*	0.61*	0.72*	0.77*	0.81*	0.81*	
∑PAHs	0.79*	− 0.04	− 0.06	0.77*	0.96*	0.83*	0.99*	0.99*	0.98*	0.98*	0.78*
C_OM_	0.79*	− 0.11	− 0.09	0.81*	0.73*	0.70*	0.77*	0.77*	0.75*	0.70*	0.28
TN	0.77*	0.10	0.10	0.81*	0.77*	0.75*	0.77*	0.75*	0.74*	0.72*	0.38
C/N	0.49*	− 0.33	− 0.26	0.48*	0.48*	0.43*	0.48*	0.47*	0.44*	0.40*	0.11
sand	0.00	0.17	0.20	0.03	− 0.16	− 0.07	− 0.25	− 0.27	− 0.31	− 0.29	− 0.35
silt	0.22	− 0.11	− 0.14	0.16	0.32	0.16	0.28	0.28	0.23	0.25	0.13
clay	− 0.14	− 0.13	− 0.16	− 0.14	0.12	− 0.18	0.13	0.17	0.25	0.22	0.51*

^*^Statistically significant at *p* < 0.05

### PCA analysis

**Fig. 4:**
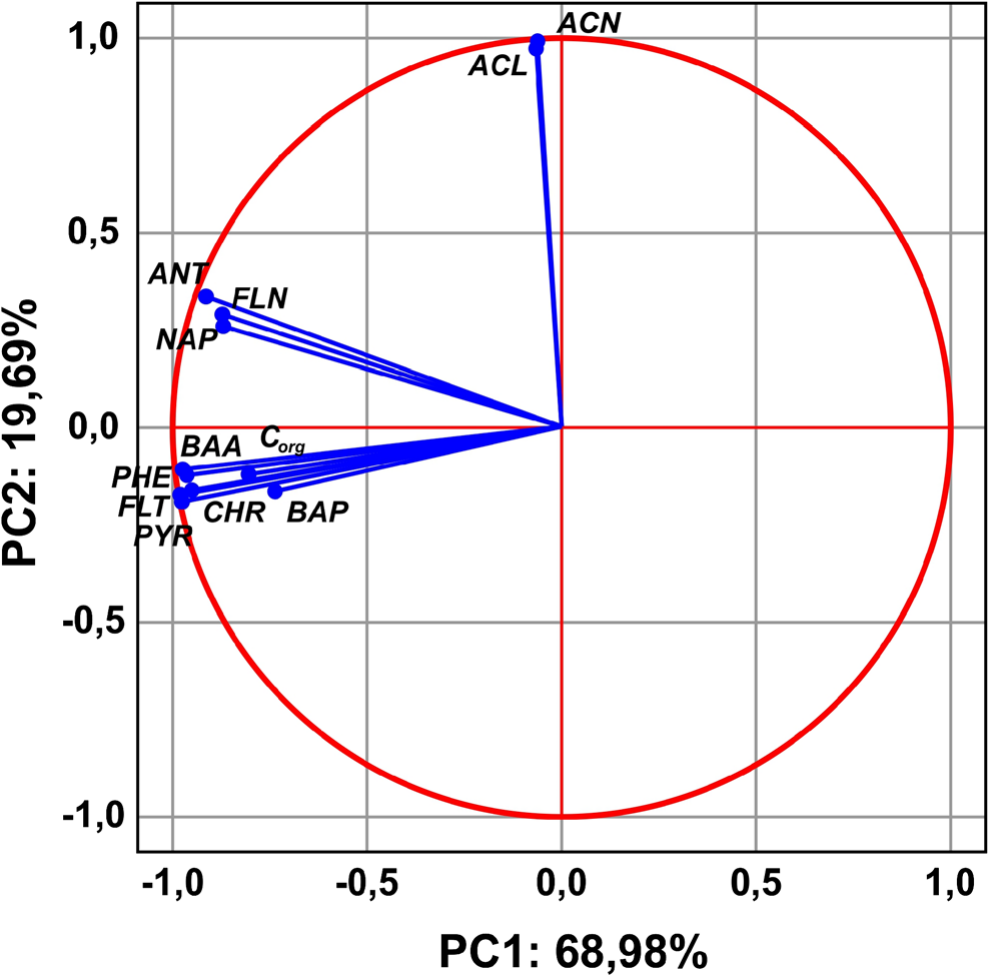
Results of PCA relationships between PAHs in the bottom sediments

**Fig. 5:**
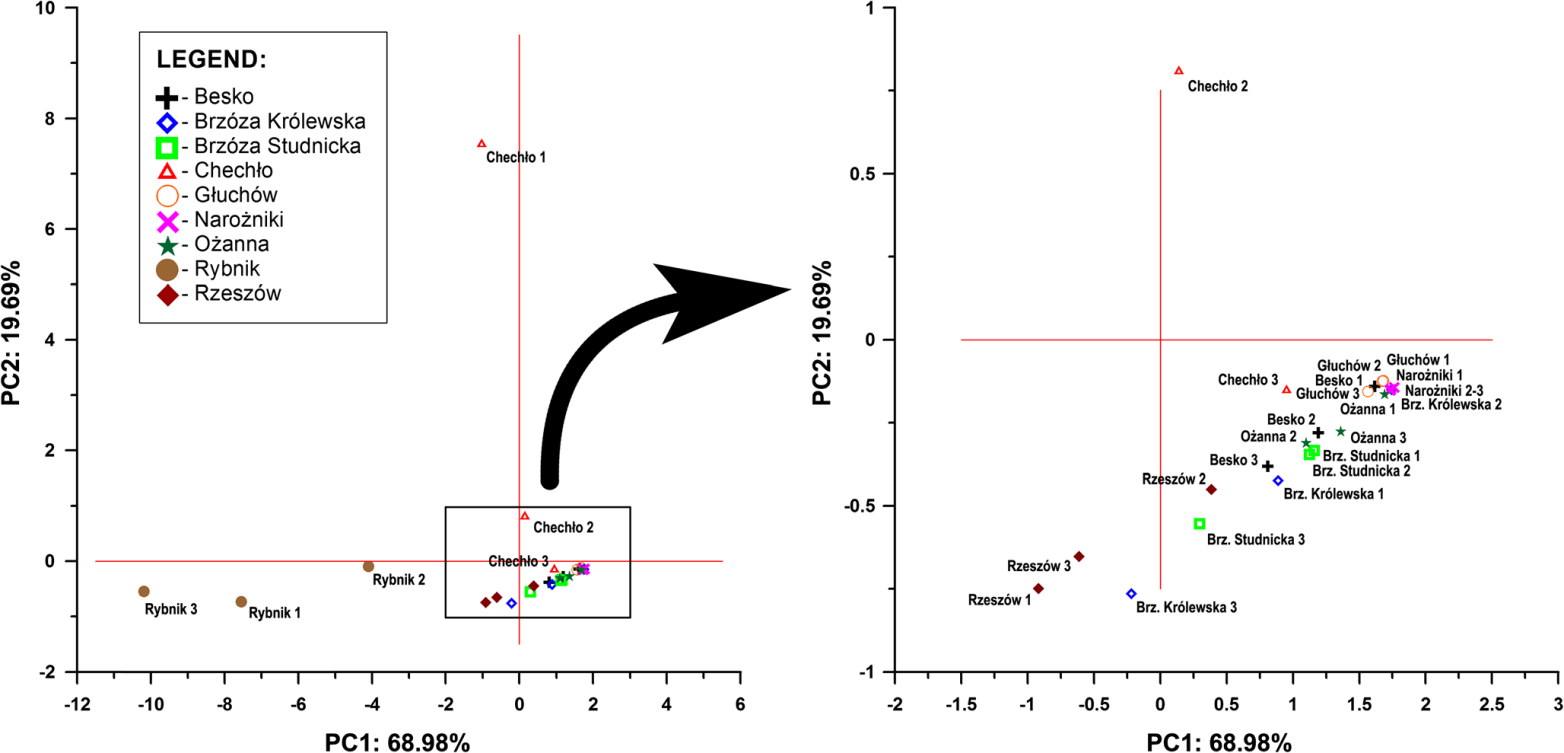
Factor loadings obtained as a result of PCA of PAHs in sediments collected from the nine reservoirs

The conducted analysis of the main components partially confirmed the previous observations. There were two distinct groups of compounds strongly correlated with each other (Fig. 4). The first and most numerous group includes such compounds as: BAA, PHE, CHR, FLT, PYR, BAP and organic carbon. The second and less numerous group consists of ANT, FLN and NAP. The third group consists of ACL and ACN. Here, it should be also reminded that compounds forming the third group are weakly represented in the whole data set, due to the fact that they were not found in most of the analyzed sediment samples from the reservoirs. The first group and the second group, although they form separate groupings on the chart, also remain positively correlated with each other, but it is not as strong a correlation as in the case of intergroup parts. The third group (two compounds) does not show any correlation, positive or negative, with the two previous groups. Additionally, as the chart and the projection of variables on the plane show, the first two groups are, in their entirety, part of the structure (they define the variance) of the first factor, defining in total almost 70% of total variance of the studied variables (Table 7). The third group of variables is responsible for a small percent of variance in the second factor. The first two primary factors define over 88% of total variance in the set of results analyzed. PCA analysis also confirmed the previously observed slight difference between the reservoirs in the profile of variables analyzed. Most points, located on a plane defined by the first two primary factors, are focused on a small point near the center of the coordinate system. Only the points representing the Rybnik and Chechło reservoirs differ considerably from this grouping (Fig. 5). Among the other reservoirs, only the one in Rzeszów slightly differs from the rest, whereas successive differences might be investigated only as micro-differences. Using these features as guidelines, it can be said that in terms of chemical state, the sediments (taking into account the PAHs analyzed and organic carbon) differ only slightly from one another, even showing a similar (apart from the Rybnik and Chechło reservoirs) characteristic.

**Table 7:** Component matrix for variables

Variables	PCA 1	PCA 2
NAP	0.825	0.351
ACL	− 0.019	0.953
ACN	− 0.027	0.979
FLN	0.826	0.392
PHE	0.960	0.003
ANT	0.866	0.451
FLT	0.993	− 0.068
PYR	0.996	− 0.086
BAA	0.994	− 0.024
CHR	0.980	− 0.085
BAP	0.764	− 0.127
C_OM_	0.766	0.040
% of the total variance	68.98	19.69

## Discussion

The bottom of the dam reservoirs is a place of sedimentation of organic and mineral matter as well as pollutants. The structure of the sediment makes them a natural geosorbent, in which pollutants are introduced to the aquatic environment. Moreover, fast rate of accumulation of these substances transported by river and flowing off the drainage area often causes their degradation and silting-up. Reservoir bottom sediments are therefore an important source of information about the aquatic environment anthropopressure, whereas their chemical composition is an important indicator of geochemical situation in the river catchment. At the same time, it should be emphasized that quantitative aspect of silting of reservoirs and qualitative aspect of bottom sediments are increasingly gaining importance because of the undisputed direct relationship between the quality of bottom sediments and the state of water in reservoirs. Considering bottom sediment pollutants, PAH concentration has a significant share, which in some determined concentrations are characterized by harmfulness and toxicity for living as well as ability to enter food chains. The studies of Dmitruk et al. (2013) showed that the mean concentration of PAHs in bottom sediments of dam reservoirs (15 reservoirs) in southern and central Poland varied from 0.1 to 7459 μg kg^−1^ DM. On the other hand, Jancewicz et al. (2015), when analyzing PAH concentration in bottom sediments from six dam reservoirs, showed that the total PAH concentration was between 24 and 12,103 μg kg^−1^ DM. In sediments of the south-eastern part of the Baltic Sea, in the Gulf of Gdansk, the total of Σ12 PAH concentrations ranged from 9 to 5100 μg kg^−1^ DM (Bartkowski et al. 2016). Hiller et al. (2009) found that concentrations of ∑16 PAHs in bottom sediments from three water reservoirs from the Slovak Republic were between 84 and 29,538 μg kg^−1^ DM. In other studies, concentrations of ∑16 PAHs in surface sediments of Fenhe reservoir and watershed of China ranged from 539.0 to 6281.7, with the mean of 2214.8 μg kg^−1^ DM (Li et al. 2015). The concentration of the sum of 17 PAHs in the bottom sediments from Wisłoka river (south-eastern part of Poland) ranged from 218 to 8437 μg kg^−1^ of DM (Książek et al. 2016). Our study showed a higher concentration of PAHs in sediments, and total mean concentration of PAHs in all surface sediments was 4933 μg kg^−1^ DM (150–33,900 μg kg^−1^ DM). In the study of Dmitruk et al. (2013), BAA, BBF and BAP were predominant compounds. In our study among all 11 PAHs, BAP, FLT and PYR were the major compounds of total PAHs in the analyzed sediments. The highest concentrations of total PAHs were shown in sediments from the Rybnik (13260–33,900 μg kg^−1^ DM)—Tables 3 and 4. The Rybnik reservoir is located in the center of the Rybnik Coal Region, one of the main industrial centers of Poland. The mining and energy industry, metallurgical and food industries dominate in the area of Rybnik. In addition, the Rybnik is a heavily urbanized and industrialized city, which is connected with a large anthropopression of the natural environment (Baran and Tarnawski 2015; Baran et al. 2016). To reservoir contamination is also caused by the Rude and Nancy rivers which flows into the reservoir. Therefore, it can be assumed that contaminated wastewater loading of the reservoir is linked from the direct impact of the industrial contamination caused by the Rybnik Coal Region and long-range transport from the Upper Silesian Industrial. Due to the high concentration of the industry, Rybnik is the third city in Poland in terms of the emission of gaseous contaminants and the fifth city in terms of the amount of industrial solid waste produced (Loska and Wiechuła 2003).

This study also showed a high concentration of PAHs in the bottom sediments of the Rzeszow reservoir (Tables 3, 4). The reservoir is under strong human pressure associated with industry, transport and local agriculture that causes severe erosion of the land, as a result of depositing the rubble and diffuse pollution (Koniarz et al. 2015a; Bartoszek et al. 2015). Bartoszek et al. (2015) showed that the greatest impact on the quality of bottom sediments is connected with the presence of BAP. Concentrations of BAP in the obtained samples were within 60.5–143.6 μg kg^−1^ DM and did not exceed values of PEC (Bartoszek et al. 2015). Our study also showed that BAP was the major compound in total PAHs in sediments from the Rzeszów reservoir—Table 4. The mean concentration of BAP in sediments from the Rzeszów reservoir was 1977 μg kg^−1^ DM, indicating a higher possibility of occurrence of adverse ecological effect (> PEC value). It is noteworthy that the mean concentration of BAP noted for 150 Polish lakes was 315 μg kg^−1^ DM (Bojakowska et al. 2012). In this study, the mean concentration of BAP was 2.5-fold higher in comparison with those of the sediments from Polish lakes. In the bottom sediment of the Besko reservoir, located in the top section of the Wislok river about 40 km above the Rzeszów reservoir, 693 μg kg^−1^ of BAP was detected, which accounted for almost 71% of the maximum value in the Rzeszów reservoir. There is a risk that during high water or flood, those deposits will be washed away and transported down the river (Bartoszek et al. 2015). In order to identify the relationships between PAHs and clay content in sediments from the Rzeszów and Besko reservoirs, Pearson’s correlation coefficient was used. Statistical analyses revealed a positive correlation between content of PAHs and fine-grained fraction of sediments (*r* = 0.93 to 0.98, *p* < 0.05). However, in the sediment from the Besko reservoir, correlation analysis revealed a negative correlation between content of PAHs and fine-grained fraction of sediments (*r* = − 0.27 to 0.95, *p* < 0.05). We think that the difference is due to the degree of silting of both reservoirs. In the Rzeszów reservoir, measurements conducted after 14 years of the reservoir operation revealed its diminished capacity by ca. 60%. However, in the Besko reservoir, bottom materials are deposited mainly in the backwater zone, making it very shallow and total loss of capacity does not exceed 10%. The silting process is responsible for the inflow of fine (mineral or organic) fractions of both natural and anthropogenic origin.

## Conclusions

In summary, our study demonstrated that the total PAH concentration in the bottom sediments differs only slightly from one another, even showing a similar characteristic (except Rybnik and Chechło reservoirs). The concentration of PAHs decreased in the following order: Rybnik Brzóza Królewska Brzóza Stadnicka Besko Chechło Ożanna Głuchów Narożniki. The major inputs of PAHs are of pyrolytic origin. However, petrogenic sources of PAHs occur especially in the Chechło and Głuchów reservoirs. The assessment of potential ecological risk indicated that non-adverse effects on the benthic fauna may occur for sediments from the Głuchów, Narozniki and Ożanna reservoirs, while slightly adverse effects were found for sediments from the Brzóza Królewska, Besko, Brzóza Stadnicka and Chechło reservoirs. The other sediments showed moderate (Rzeszów reservoirs) and strong effect (Rybnik reservoir) on biological communities. PCA analysis found slight difference between the reservoirs in the profile of variable PAHs. Most points, located on a plane defined by the first two primary factors, are focused on a small point near the center of the coordinate system. Only the points representing the Rybnik and Chechło reservoirs differ considerably from this grouping.
